# Supplementary material for: An intralayer microcircuit in the temporal association cortex underlies sensory-induced escape in mice
Source: Nat Commun. 2026 Mar 17;17:4088. doi: 10.1038/s41467-026-70754-z (PMC13144386; doi:10.1038/s41467-026-70754-z)
Supplement: Supplementary file 1 — Supplementary Information [file 41467_2026_70754_MOESM1_ESM.pdf]

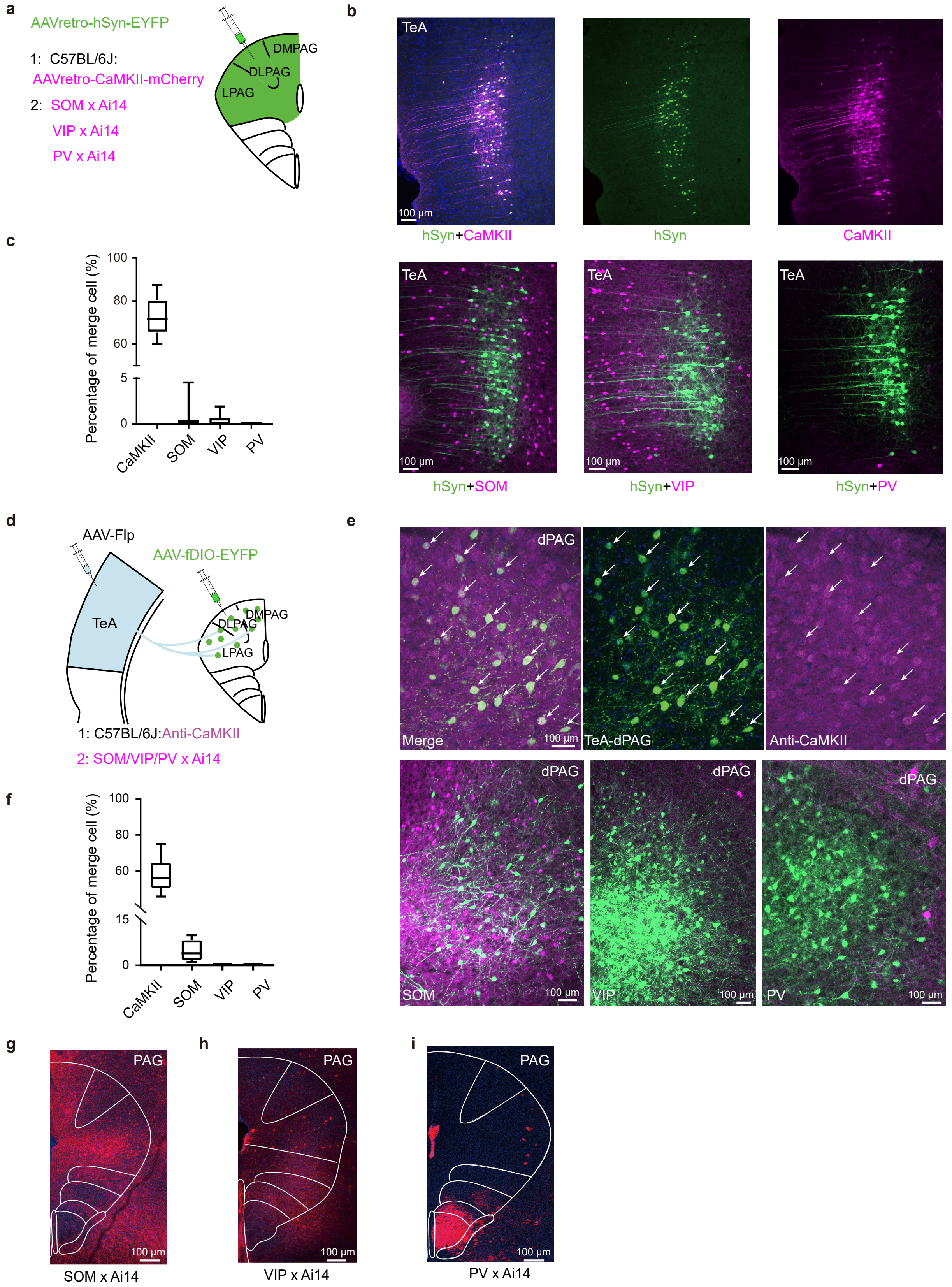

Supplementary Fig. 1 | Cells in the TeA and dPAG that constitute the TeA–dPAG circuit.

a Labelling of TeA_{dPAG} neurons via injections of AAVretro-hSyn-EYFP with AAVretro-CaMKII-mCherry in the dPAG of wild-type mice (1) or Cre transgenic mice (*SOM-Cre*, *Vip-Cre*, and *PV-Cre* mice crossed with *Ai14* mice) (2). **b** Images showing mCherry- (expression in magenta) and EYFP-labelled TeA_{dPAG} neurons in wild-type mice (top) and Cre transgenic mice (bottom). Ai 14 reporter expression is shown in magenta. White indicates overlapping labelling in the neurons. **c** Percentages of retrogradely labelled TeA_{dPAG} hSyn neurons overlapping with CaMKII, SOM, VIP, or PV labelling; n = 18, 22, 21, and 25 slices from 5, 4, 4, and 5 animals, respectively. **d** Schematic of virus injection and experimental protocol. Sections from wild-type mice were stained with an anti-CaMKII antibody (1), and *SOM-Cre*, *Vip-Cre*, and *PV-Cre* mice were crossed with *Ai14* mice (2). **e** Co-localization of hSyn-EYFP-labelled $_{TeA}$dPAG neurons with anti-CaMKII (top) or SOM, VIP, and PV Ai14 neurons (bottom). mCherry and Ai 14 reporter expression is shown in magenta. **f** Percentages of EYFP-labelled $_{TeA}$dPAG neurons overlapping with anti-CaMKII, SOM, VIP*,* and PV neurons; n = 9, 8, 7, and 7 slices from 4, 3, 4, and 4 animals, respectively. **g-i** (**g**) SOM, (**h**) VIP, and (**i**) PV neurons in the PAG. Unless specified, Box plots show the upper and lower quartiles, with whiskers indicating the min and max values, and the center line representing the mean. (Source data are provided as a Source Data file).

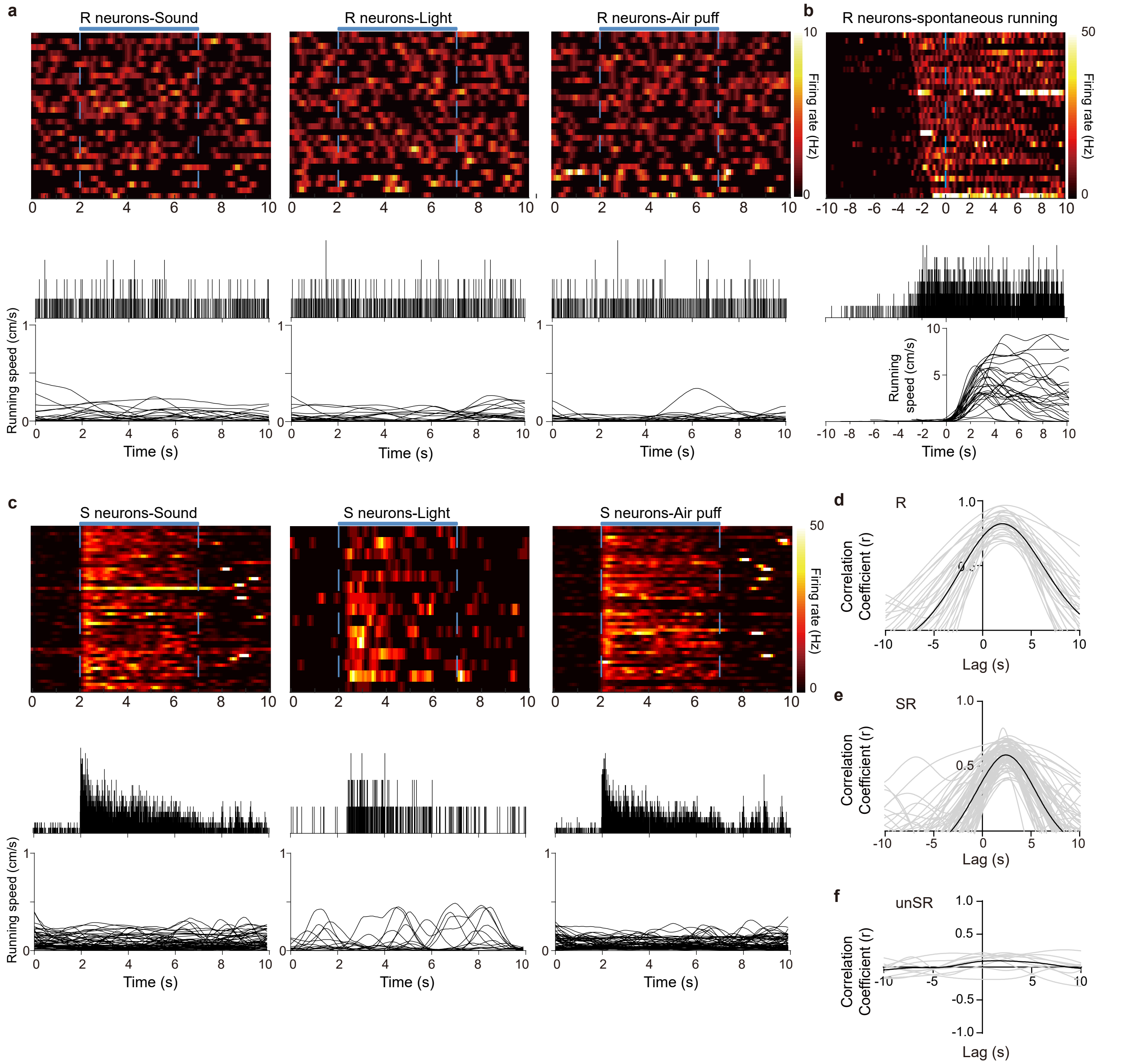

Supplementary Fig. 2 | S- and R-neurons in the TeA.

a Responses of the R-neurons to three sensory stimuli. Top: trial-averaged activity heatmaps for each neuron, aligned to stimulus onset (2 s) and excluding trials with movement (> 0.5 cm/s). Each row represents the averaged activity of a single R-neuron across all trials. (n = 29 neurons for sound, light and air puff, respectively). Middle, population PSTHs. Bottom, running speed aligned to stimulus onset for each neuron (each trace is the average across all trials presented during that neuron's recording; n = 29 neurons for sound, light, air puff, respectively). Blue bars: 5 s stimulus duration. **b** Activity of R-neurons during spontaneous running (no sensory stimuli). Data from 29 neurons. Top,heatmap of firing rates aligned to running onset (0 s). Each row represents the averaged activity of a single R-neuron across all spontaneous movement trials. Blue dashed lines indicate alignment to running onset (0 s). Middle, population PSTH aligned to running onset. Bottom, running speed aligned to running onset for each neuron (each trace is the average across all trials presented during that neuron's recording; n = 29 neurons). **c** Responses of S-neurons (n = 75; 66 SR, 9 unSR) to sound, light, and air puff stimuli. Neurons responsive to each: 51, 15, 50. Top: trial-averaged activity heatmaps for each neuron (aligned to onset, 2 s; no-running trials). Each row represents the averaged activity of one S-neuron. Middle: population PSTHs. Bottom: running speed aligned to each stimulus onset for each neuron (each trace is the average across all trials presented during that neuron's recording). Blue bars: 5 s stimulus duration. **d–f** CCF analyses of R-neurons (**d**, n = 29 neurons), SR-neurons (e, n = 66 neurons) and unSR-neurons (**f**, n = 9 neurons). The black line represents the mean CCF curve; grey lines indicate individual neuronal CCF traces. (Source data are provided as a Source Data file).

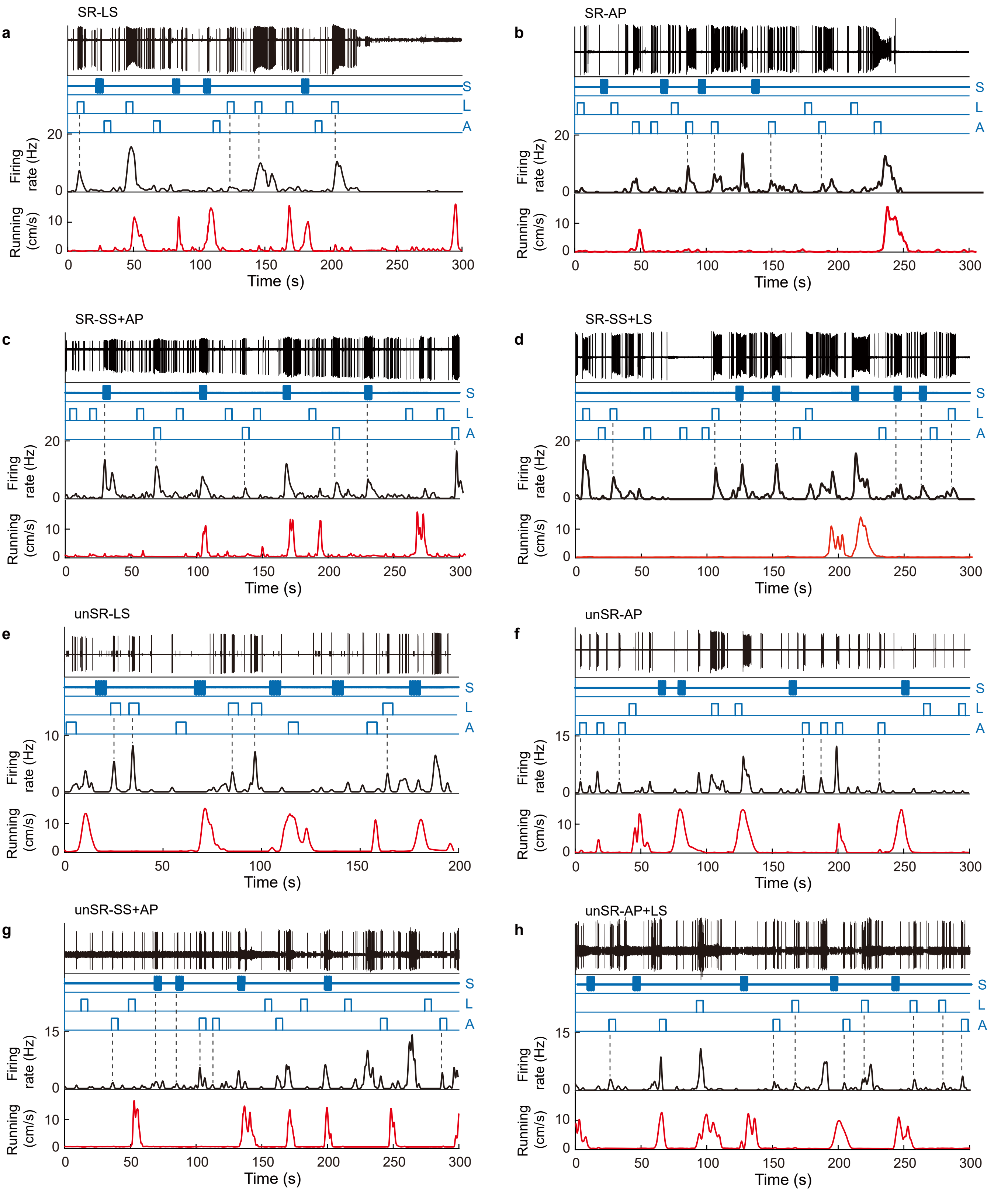

Supplementary Fig. 3 | SR-neurons and unSR-neurons respond to different sensory stimuli.

a–d Representative firing patterns of SR-neurons in response to light stimulation (LS) (**a**), air puff stimulation(AP) (**b**), sound and air puff stimulation (SS+AP) (**c**), sound and light stimulation (SS+LS) (**d**) related to running speed (red). The black dashed lines indicate events that can be used to analyze the correlation between neuronal firing and sensory stimuli, where the sensory stimuli did not induce running. **e–h** Representative firing patterns of unSR-neurons in response to LS (**e**), AP (**f**), SS+AP (**g**), and air puff and light stimulation (AP + LS) (**h**) unrelated to running speed (red). A representative SR-neuron that responded to SS is shown in **Fig. 4h**. A representative unSR-neuron that responded to SS is shown in **Fig. 4m**.

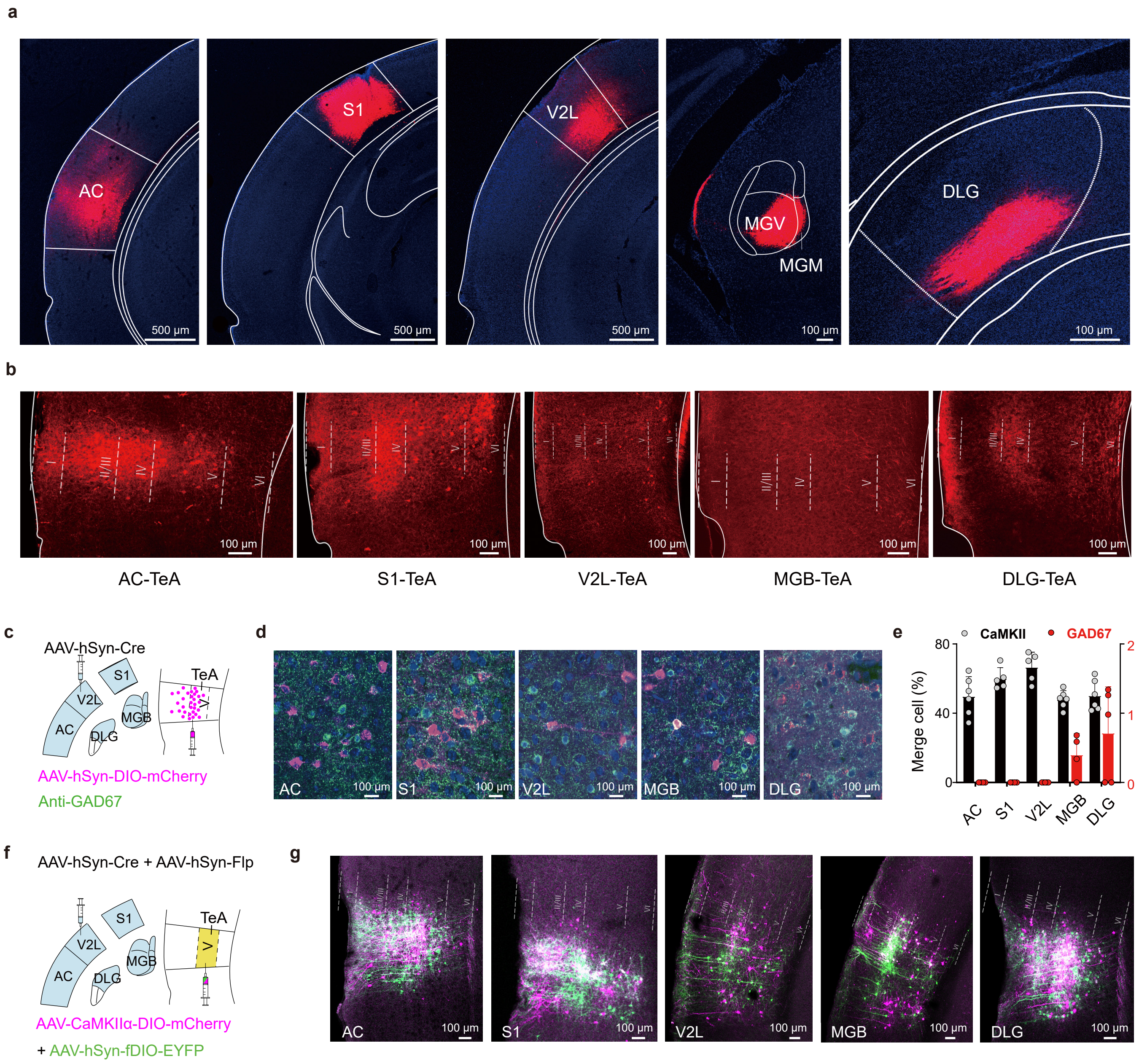

Supplementary Fig. 4 | Main source regions projecting to the TeA.

a Sites of AAV2/9-hSyn-mCherry injection into the AC, S1, V2L, MGB, and DLG. **b** Distribution of axons from different source regions within the TeA. **c, f** Schematic of virus injection and experimental protocol. **d** Images showing hSyn (mCherry, expression in magenta) and anti-GAD 67-labelled (EYFP) neurons. **e** Percentages of neurons with overlapping labelling with anti-GAD 67 in (**d**) and with CaMKII in (**g**). Anti-GAD 67 labelling: n = 4, 4, 5, 4, and 5 slices from 3, 4, 3, 3, and 3 animals.CaMKII labelling: n = 6, 5, 5, 6, and 6 slices from 4, 4, 3, 4, and 4 animals. **g** Images showing mCherry- and EYFP-labelled $_{\text{Sens}}$TeA neurons. Data are presented as mean ± SD. (Source data are provided as a Source Data file).

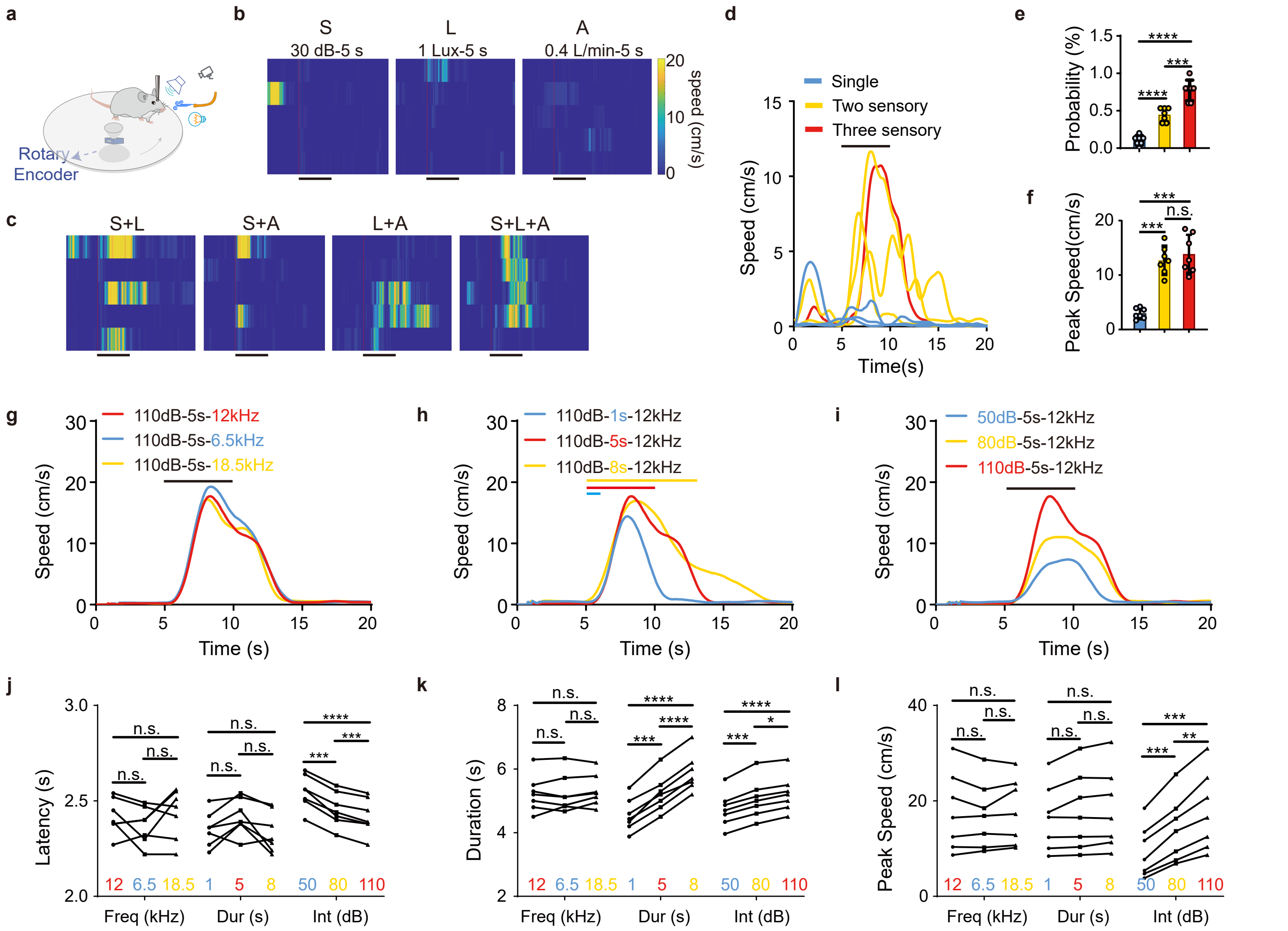

Supplementary Fig. 5 | Multisensory integration.

a–f Paradigm of the running model (**a**) with subthreshold or suprathreshold unisensory (**b**, sound: 30 dB, 12 k; light: 1 lux and air puff: 0.4 L/min) or multisensory stimuli in different combinations for inducing running shown as heatmaps (**c**) and running speed traces (**d**, 5 trials) and corresponding plots of the probability of running (**e**) and peak speed (**f**). (n = 7 animals, ****P* < 0.001, *****P* < 0.0001, one-way RM ANOVA with the Bonferroni post hoc correction). **g-i** Average speed (10 trials) in response to sounds with different frequencies (**g**, in kHz), durations (**h**, in s), and intensities (**i**, in dB SPL). **j-l** Latency (**j**), duration (**k**), and peak speed (**l**) of running induced by sound stimuli of different frequencies (Freq), durations (Dur), and intensities (Int). (n = 7 animals, **P* < 0.05, ***P* < 0.01, ****P* < 0.001, *****P* < 0.0001, one-way RM ANOVA with the Bonferroni post hoc correction). Data are presented as mean ± SD. (Source data are provided as a Source Data file; see Supplementary Data 1 for detailed statistics).

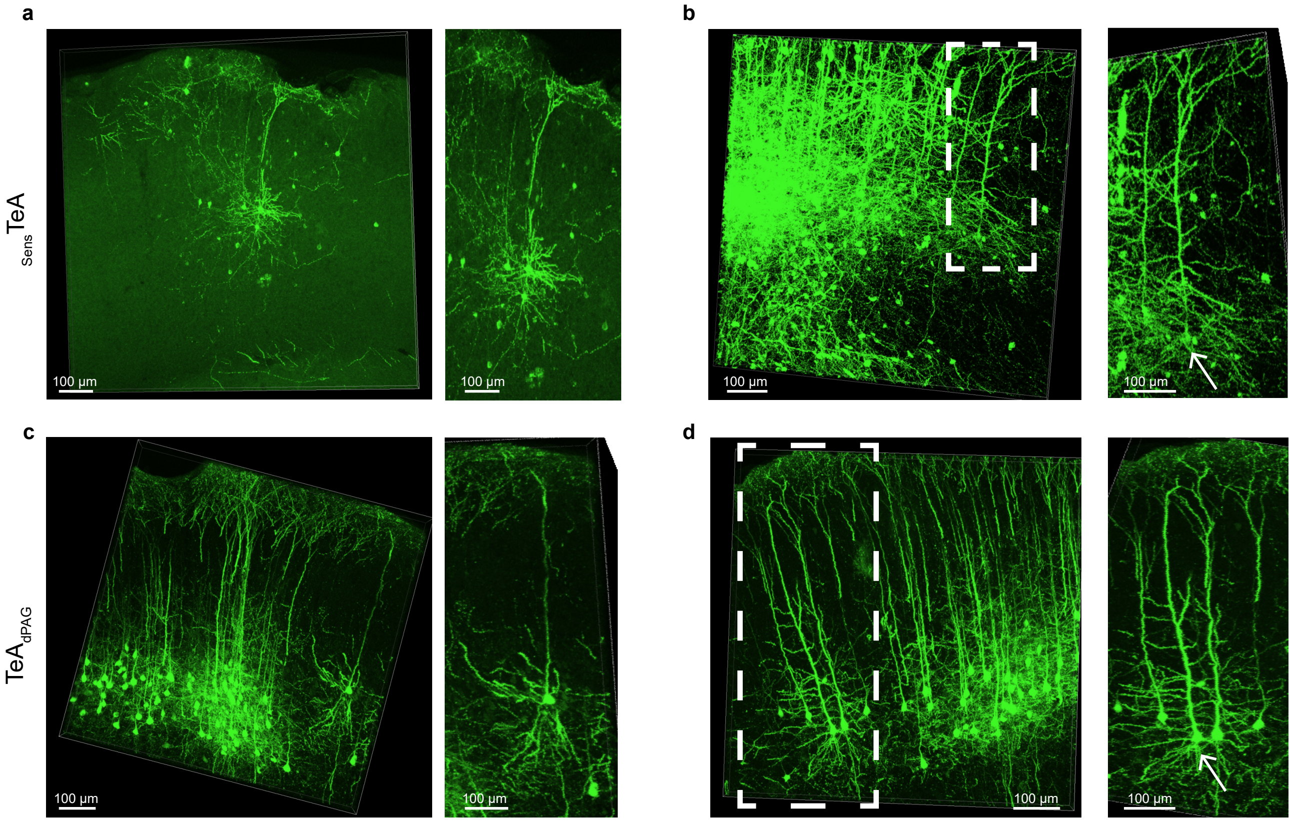

Supplementary Fig. 6 | Three-dimensional reconstructed images showing the morphology of individual $_{Sens}$TeA and TeA$_{dPAG}$ neurons.

a 3D reconstruction of an individual labeled $_{Sens}$TeA neuron (left) and a magnified view highlighting its morphological details (right). **b** 3D reconstruction of $_{Sens}$TeA neurons in a region with higher viral labeling density (left). The white dashed area indicates a sparsely labeled region selected for morphological reconstruction. The neuron used for reconstruction is marked by a white arrow (right). **c, d** Same as (**a**) and (**b**), respectively, but shown for TeA$_{dPAG}$ neurons.
