# Supplementary material for: An intralayer microcircuit in the temporal association cortex underlies sensory-induced escape in mice
Source: Nat Commun. 2026 Mar 17;17:4088. doi: 10.1038/s41467-026-70754-z (PMC13144386; doi:10.1038/s41467-026-70754-z)
Supplement: Supplementary file 2 — Description of Additional Supplementary Files [file 41467_2026_70754_MOESM2_ESM.pdf]

Description of Additional Supplementary Files

Supplementary Data 1

Statistical source data and detailed p-values for all analyses reported in the manuscript.

Supplementary Movie 1

The merged neurons (yellow) shown in Fig. 6h.
