# Supplementary material for: An intralayer microcircuit in the temporal association cortex underlies sensory-induced escape in mice
Source: Nat Commun. 2026 Mar 17;17:4088. doi: 10.1038/s41467-026-70754-z (PMC13144386; doi:10.1038/s41467-026-70754-z)
Supplement: Supplementary file 5 — Reporting Summary [file 41467_2026_70754_MOESM5_ESM.pdf]

nature portfolio

Corresponding author(s): Wen Zhong & Zhongju Xiao

Last updated by author(s): Feb 15, 2026

Reporting Summary

Nature Portfolio wishes to improve the reproducibility of the work that we publish. This form provides structure for consistency and transparency in reporting. For further information on Nature Portfolio policies, see our Editorial Policies and the Editorial Policy Checklist.

Statistics

For all statistical analyses, confirm that the following items are present in the figure legend, table legend, main text, or Methods section.

n/a	Confirmed	
☐	☒	The exact sample size (*n*) for each experimental group/condition, given as a discrete number and unit of measurement
☐	☒	A statement on whether measurements were taken from distinct samples or whether the same sample was measured repeatedly
☐	☒	The statistical test(s) used AND whether they are one- or two-sided<br>*Only common tests should be described solely by name; describe more complex techniques in the Methods section.*
☐	☒	A description of all covariates tested
☐	☒	A description of any assumptions or corrections, such as tests of normality and adjustment for multiple comparisons
☐	☒	A full description of the statistical parameters including central tendency (e.g. means) or other basic estimates (e.g. regression coefficient) AND variation (e.g. standard deviation) or associated estimates of uncertainty (e.g. confidence intervals)
☐	☒	For null hypothesis testing, the test statistic (e.g. F, t, r) with confidence intervals, effect sizes, degrees of freedom and P value noted<br>*Give P values as exact values whenever suitable.*
☒	☐	For Bayesian analysis, information on the choice of priors and Markov chain Monte Carlo settings
☒	☐	For hierarchical and complex designs, identification of the appropriate level for tests and full reporting of outcomes
☒	☐	Estimates of effect sizes (e.g. Cohen's *d*, Pearson's *r*), indicating how they were calculated

Our web collection on statistics for biologists contains articles on many of the points above.

Software and code

Policy information about availability of computer code

Data collection	1. Signals (spikes, excitatory postsynaptic current, and inhibitory postsynaptic current) were recorded using a MultiClamp 700B amplifier (Axon, USA), BrainWare 32 (Tucker-Davis Technologies, USA) and Clampex 10.2 software (Axon, USA).<br>2. The speed signals and video were recorded using USD Device Explorer software (US Digital, USA) and Windows Camera software (Microsoft, USA).<br>3. The mouse's behavior was monitored and recorded using an external high-definition infrared camera (LRCP10620, 20 FPS, China).<br>4. Coronal sections were cut using a cryostat (Leica CM1860, Germany).<br>5. Fluorescence signals were visualized using a laser scanning confocal microscope (A1R, Nikon).
Data analysis	1. Excel 2016, OriginPro 2017 (OriginLab Corporation), MATLAB 2016b (MathWorks),BrainWare 32 (Tucker-Davis Technologies, USA) and Clampfit 10.2 software (Axon, USA) were used to export and analyze the recorded electrophysiological data.<br>2. VisuTrack software was used to analyzed the data of the open field test.<br>3. ImageJ 1.4 (NIH) or NIS Elements software were used to analyzed fluorescence signals.<br>4. Cross-correlation function analysis between the running curve and neuronal firing curve was performed using SPSS software (SPSS 21, IBM).<br>5. Statistical analysis was performed using SPSS (SPSS21, IBM).<br>6. OriginPro 2017 (OriginLab Corporation) or GraphPad Prism 10 (GraphPad Software) were used for statistical analysis and graphing.<br>7. The software used for animal running speed and neuron firing rate data analysis is open source and available on GitHub (https://github.com/LiHe0606/Animal-speed-and-Neuron-firing-1-XiaoLab.).

For manuscripts utilizing custom algorithms or software that are central to the research but not yet described in published literature, software must be made available to editors and reviewers. We strongly encourage code deposition in a community repository (e.g. GitHub). See the Nature Portfolio guidelines for submitting code & software for further information.

Data

Policy information about availability of data

All manuscripts must include a data availability statement. This statement should provide the following information, where applicable:

- Accession codes, unique identifiers, or web links for publicly available datasets
- A description of any restrictions on data availability
- For clinical datasets or third party data, please ensure that the statement adheres to our policy

All of the raw data for behavioral, electrophysiological, and immunohistochemical analyses are provided in the main text or supplementary materials and source data file.

Research involving human participants, their data, or biological material

Policy information about studies with human participants or human data. See also policy information about sex, gender (identity/presentation), and sexual orientation and race, ethnicity and racism.

Reporting on sex and gender	n/a
Reporting on race, ethnicity, or other socially relevant groupings	n/a
Population characteristics	n/a
Recruitment	n/a
Ethics oversight	n/a

Note that full information on the approval of the study protocol must also be provided in the manuscript.

Field-specific reporting

Please select the one below that is the best fit for your research. If you are not sure, read the appropriate sections before making your selection.

[x] Life sciences [ ] Behavioural & social sciences [ ] Ecological, evolutionary & environmental sciences

For a reference copy of the document with all sections, see nature.com/documents/nr-reporting-summary-flat.pdf

Life sciences study design

All studies must disclose on these points even when the disclosure is negative.

Sample size	The sample size was based on the variation of the measure, and the requirement for statistical tests.
Data exclusions	Animals that failed to maintain such locomotor stability (5%), characterized by limb coordination deficits or complete immobility, were excluded from subsequent recordings and analysis to ensure experimental consistency.
Replication	The reproducibility of all experiments is confirmed; key results were validated through a minimum of three independent biological replicates.
Randomization	Animals were numbered and randomly divided into groups according to a random number table.
Blinding	All the investigators were blinded too group allocation during data collection and analysis.

Reporting for specific materials, systems and methods

We require information from authors about some types of materials, experimental systems and methods used in many studies. Here, indicate whether each material, system or method listed is relevant to your study. If you are not sure if a list item applies to your research, read the appropriate section before selecting a response.

Materials & experimental systems

n/a	Involved in the study
☐	☒ Antibodies
☒	☐ Eukaryotic cell lines
☒	☐ Palaeontology and archaeology
☐	☒ Animals and other organisms
☒	☐ Clinical data
☒	☐ Dual use research of concern
☒	☐ Plants

Methods

n/a	Involved in the study
☒	☐ ChIP-seq
☒	☐ Flow cytometry
☒	☐ MRI-based neuroimaging

Antibodies

Antibodies used	1. Anti-CaMKII monoclonal rabbit antibody (1:200; Abcam, USA, Catalog # ab52476).<br>2. Anti-GAD67 monoclonal mouse antibody (1:200; Millipore, USA, Catalog # MAB5406)<br>3. Streptavidin-Cy3 (1:200; Thermo Fisher Scientific, USA, Catalog # 438315)<br>4. Goat anti-Rabbit IgG Alexa Fluor 647 (1:500; Invitrogen™, USA, Catalog # A21244,)<br>5. Goat anti-Mouse IgG (H+L) Cross Adsorbed Secondary Antibody, Alexa Fluor 488 (1:500; Invitrogen™, USA, Catalog # A32723)
Validation	1. Anti-CaMKII monoclonal rabbit antibody: https://www.abcam.cn/products/primary-antibodies/camkii-antibody-ep1829y-ab52476.html<br>2. Anti-GAD67 monoclonal mouse antibody (MAB5406; Millipore, USA): https://www.sigmaaldrich.cn/CN/zh/product/sigma/zms5406?utm_campaign=LR4_696_ZooMab+1+-+China&utm_medium=cpc&utm_source=bing&utm_content=sigma 2Fzms5406&utm_term=gad67%E6%8A%97%E4%BD%93&msclkid=60a47ab962091b36f54a1db1a8c961df<br>3. Streptavidin-Cy3:https://www.thermofisher.cn/order/catalog/product/438315<br>4. Goat anti-Rabbit IgG Alexa Fluor 647:https://www.thermofisher.cn/cn/zh/antibody/product/Goat-anti-Rabbit-IgG-H-L-Cross-Adsorbed-Secondary-Antibody-Polyclonal/A-21244<br>5. Goat anti-Mouse IgG (H+L) Cross Adsorbed Secondary Antibody, Alexa Fluor 488 (A32723, Invitrogen™, USA): https://www.thermofisher.cn/cn/zh/antibody/product/Goat-anti-Mouse-IgG-H-L-Highly-Cross-Adsorbed-Secondary-Antibody-Polyclonal/A32723

Animals and other research organisms

Policy information about studies involving animals; ARRIVE guidelines recommended for reporting animal research, and Sex and Gender in Research

Laboratory animals	Male and female C57BL/6j (Laboratory Animal Center of Southern Medical University, Guangzhou, China), SOM-Cre (RRID: IMSR_JAX: 013044), Vip-Cre (RRID: IMSR_JAX: 031628), PV-Cre (RRID: IMSR_JAX: 008069), Ai14 (Cre-dependent tdTomato reporter line RRID: IMSR_JAX: 007914) mice aged 4-12 weeks were used. All Cre driver lines were crossed to Ai14. Mice were housed in a vivarium with temperature controlled at 21-25°C and humidity at 50-60%, and 12 hours a day/night cycle (light turned on at 8 am). Food and water were available ad libitum.
Wild animals	The study did not involve wild animals.
Reporting on sex	The mice were male and female.
Field-collected samples	No field collected samples were used in this study.
Ethics oversight	All experiments were conducted in accordance with the Regulations on the Management of Laboratory Animals (China) and were approved by the Animal Ethics Committee of Southern Medical University.

Note that full information on the approval of the study protocol must also be provided in the manuscript.

Plants

Seed stocks	n/a
Novel plant genotypes	n/a
Authentication	n/a
